# Pullulan-Based Gels with Food-Related Orientation: From Microbial Production to Synergistic Assemblies

**DOI:** 10.3390/gels12070631

**Published:** 2026-07-15

**Authors:** Maria Syrigou, Erminta Tsouko

**Affiliations:** Division of Genetics & Biotechnology, Department of Biology, National and Kapodistrian University of Athens, 15784 Athens, Greece; marysyrigou28092004@gmail.com

**Keywords:** biopolymer, physicochemical resilience, agro-industrial waste, tailored properties, synergistic gelation, food gels

## Abstract

Pullulan is a microbial exopolysaccharide produced primarily by *Aureobasidium* spp. It has attracted considerable attention due to its biodegradability, biocompatibility, and versatility in food-related applications. While its basic chemical structure has long been established, recent advances have significantly improved the understanding of the genetic, enzymatic, and regulatory mechanisms governing its biosynthesis. This review discusses current knowledge on pullulan production, focusing on biosynthetic pathways, regulatory networks, and the influence of fermentation conditions on polymer yield and quality. Particular emphasis is placed on the utilization of agro-industrial residues as renewable feedstocks within a circular bioeconomy framework, as well as on downstream recovery and purification strategies. Furthermore, the physicochemical properties of pullulan and its ability to form synergistic assemblies with other biopolymers are evaluated in relation to hydrogels, edible films, active packaging, and bioactive delivery systems. By integrating microbial biotechnology, bioprocess engineering, and material science, this review provides a comprehensive overview of pullulan-based systems for food-related applications.

## 1. Introduction

The global biopolymer market is currently undergoing a transformative expansion, driven by the industrial shift toward sustainable, biodegradable and non-toxic, materials. Within this landscape, pullulan has evolved from a niche laboratory product to a cornerstone of macromolecular science, with applications across the pharmaceutical, cosmetic, and food sectors [1]. Pullulan is a microbial exopolysaccharide mainly produced by species of the genus *Aureobasidium*. In its natural ecological niche, pullulan facilitates essential survival processes, including cellular flocculation, aggregation, and surface adhesion. These inherent binding characteristics provide the foundation for the polymer’s versatile performance and have enabled its broad industrial utilization [2].

The commercial footprint of pullulan reflects its increasing industrial relevance [3]; in 2026, the global pullulan market was USD ~ 80 million and it is projected to reach ~USD 120 million by 2035 [4]. More than half of current production is directed toward edible films and oral drug delivery systems, where the polymer’s biocompatibility and safety [5] are essential requirements. Despite this growing demand, large-scale commercialization is limited by high production costs. Current prices of bulk pullulan range within USD 25–30 per kg, which are significantly higher than commodity petroleum-based plastics such as polyethylene (USD~1.2/kg) [6] or several established biopolymers like xanthan and dextran [7]. The economic feasibility of pullulan production is influenced by several technical challenges. These include the cost of synthetic fermentation substrates, high broth viscosity during cultivation, oxygen transfer limitations, and reduced product yields at industrial scale [7,8]. Furthermore, the co-synthesis of melanin pigments necessitates energy-intensive purification and decolorization steps, to meet food- and pharmaceutical-grade specifications [9,10].

From a material perspective, pullulan has attracted considerable attention in food-related applications due to its biodegradability, biocompatibility, transparency, and excellent film-forming capacity. It has been explored as a functional ingredient, stabilizer, thickening agent, edible coating, packaging material and bioactive delivery matrix [11]. Although pullulan does not readily form thermoreversible gels at low concentrations, its abundant hydroxyl (-OH) groups enable the formation of robust three-dimensional matrices. These structures can be generated through chemical crosslinking, physical entanglement, or synergistic interactions with proteins and polysaccharides [9,12]. The native polymer presents several limitations, including high hydrophilicity, and relatively poor moisture barrier properties. To overcome these drawbacks, hybridization, crosslinking, and nanoemulsion incorporation have been investigated, resulting in multifunctional pullulan-based materials with enhanced physicochemical and functional properties [13,14].

The functionality and industrial viability of pullulan are influenced by the underlying genetic regulation within *Aureobasidium* spp. The interplay between core biosynthetic genes (such as *UGP1* and *AmAGS2*) and regulatory transcription factors (like *NsdD* and *Msn2*) determines critical macromolecular attributes, including molecular weight, chain architecture, and pigment purity. These biological controls serve as the primary determinants of the polymer’s viscosity and mechanical resilience [15]. Consequently, current research focuses on two complementary directions. The first aims to improve production economics through the utilization of low-cost agro-industrial residues within a circular bioeconomy framework [16]. The second seeks to enhance polymer functionality through strain engineering and the development of hybrid or crosslinked materials with improved mechanical and barrier resilience [6,17].

Several reviews have examined pullulan from the perspective of its physicochemical properties, production processes, or industrial applications. At the same time, recent advances in biosynthetic regulation, renewable feedstock utilization, and the development of pullulan-based gels and hybrid materials have expanded knowledge across multiple disciplines. As a result, there is a need for an integrated assessment that connects microbial biosynthesis and bioprocess engineering with polymer structure, gel-forming behavior, and food-related applications. Therefore, the primary objective of this review is to provide a comprehensive overview of pullulan, from its microbial synthesis to its application as a functional structural matrix in food systems. Particular emphasis is given to the biosynthetic pathways and regulatory mechanisms of *Aureobasidium* spp. and their influence on polymer architecture and molecular weight. Fermentation strategies and downstream processing approaches are critically discussed, with special attention given to the valorization of agro-industrial residues. Furthermore, the mechanisms governing synergistic gel formation are examined, highlighting interactions between pullulan, proteins, and polysaccharides and their impact on the development of functional gel systems. By integrating recent advances in microbial biotechnology, bioprocess engineering, and material science, this review provides a comprehensive perspective on the factors governing the production, structure, and functionality of pullulan-based materials for food-related applications.

## 2. Biosynthetic Pathway and Genetic Regulation of Pullulan Macrostructure

### 2.1. Biosynthetic Pathway

Despite pullulan’s longstanding industrial integration, the genetic and enzymatic basis of its synthesis remained partially unverified until 2020. While its primary chemical architecture was characterized in the 1960s, the molecular blueprint for its production was only recently elucidated [18]. Historically, UDP-glucose (UDPG) was established as the fundamental precursor for pullulan synthesis. In this classic model, the metabolic supply of UDPG branches from the EMP pathway via the sequential action of α-phosphoglucomutase and UDP-glucose pyrophosphorylase which converts glucose-6-phosphate into the activated nucleotide sugar donor required for chain elongation [19,20]. While this precursor flux was well-characterized, the specific enzymes responsible for the polymerization of UDPG remained elusive for decades [18].

Recent molecular studies have elucidated the critical role of the *PUL1* gene which serves as the cornerstone of polymerization. It is proposed that the pullulan synthetase encoded by *PUL1* functions as an auxiliary protein, facilitating the activity of the core enzyme α-glucan synthetase 2 (AmAgs2) during polymer assembly [21]. This hypothesis was further supported by gene knockout experiments; the deletion of *PUL1* in *A. pullulans* resulted in the complete loss of pullulan production, with mutant strains synthesizing β-glucan [22]. Conversely, overexpression of the *PUL1* gene boosted yields by 15% compared to the wild-type and increased the polymer’s molecular mass to 4.4 × 10^5^ Da. These findings identify Pul1 as a vital regulator that interacts with the AmAgs2 synthetase to optimize both the efficiency and the structural properties of the biosynthetic process [15].

Beyond the core synthetase complex, the UDP-glucose:glycoprotein glucosyltransferase (Ugt1), encoded by the *UGT1* gene, has been identified as a significant regulator. Genetic manipulation studies on *A. melanogenum* P16 strain have demonstrated that while the deletion of the *UGT1* gene leads to reduction in pullulan yields, its overexpression has been shown to enhance production by 26.5% compared to wild-type performance [23]. Recent evidence suggests that pullulan biosynthesis is a coordinated process occurring within the cell wall and periplasmic space. Cell wall integrity, maintained by mannosyltransferases like Gt6 and Gt7, is a prerequisite for pullulan production, as its disruption suppresses the expression of key pullulan-related genes [18,24].

The assembly of the pullulan’s macromolecular chain is centrally executed by the AmAgs2 protein, an integral membrane α-glucan synthetase, encoded by the *AmAGS2* gene. Knockout and complementation studies have identified this gene as the genetic bottleneck for the entire biosynthetic pathway which [18,25] AmAgs2 orchestrates the polymer’s transition from the cytoplasm to the extracellular environment via three specialized domains. The biochemical initiation of this process shares mechanistic similarities with glycogen synthesis, relying on short α-1,4-glucan primers to facilitate polymer elongation. In *A. melanogenum* P16, these starter chains are synthesized intracellularly by specific enzymes including glycogenins (Glg1/Glg2), sterol glucosyltransferase (Sgt1), and ceramide β-glucosyltransferase (Gcs1) [21]. Nevertheless, the sustained biosynthesis of pullulan despite the inactivation of these enzymes—supported by evidence from related fungal species—indicates that intracellular α-amylases likely function as an auxiliary priming system by generating the universal α-1,4 linked glucooligosaccharides required for polymer assembly [18,26]. Following initiation, the intracellular Gys_D domain catalyzes the formation of α-1,4 glycosidic bonds within the cytoplasm to elongate the precursor chain. The transmembrane EPST_D domain transports the growing polymer across the cell membrane to the extracellular environment. Finally, the extracellular Amy_D domain hydrolyzes internal α-1,4 linkages to release maltotriose units, which are subsequently polymerized via α-1,6 glycosidic bonds to establish pullulan’s repeating structure. This coordinated transition from internal synthesis to extracellular modification ensures structural consistency and regulation of molecular weight; ultimately, this specific linkage pattern yields a linear, neutral macromolecule responsible for the unique rheological profile, mechanical strength, and viscoelasticity observed in the resulting gels [18,25].

### 2.2. Molecular and Transcriptional Control of Pullulan Biosynthesis

Beyond individual gene expression, a network of transcription factors and signaling pathways regulates the activity of these genes in response to environmental stimuli. The Msn2 transcription factor activates the *UGP1* gene and it is regulated by the cAMP-PKA pathway. High PKA activity suppresses pullulan genes to favor lipid production, whereas low PKA levels allow Msn2 to enhance synthesis [27]. Material purity is further managed by the NsdD GATA factor which serves as a molecular “switch” that activates pullulan genes while suppressing those responsible for melanin synthesis. This genetic control is essential for industrial consistency, as it ensures the high optical clarity and structural homogeneity [28], the GATA-type transcription factors (AreA/AreB regulators) relieve nitrogen catabolite repression to maximize yields [29]. Additionally, the high osmolarity glycerol pathway triggers enhanced pullulan and liamocin synthesis as a protective response to osmotic stress from high salt or sugar concentrations [28]. These regulatory systems represent vital biological drivers; by utilizing both nitrogen depletion and osmotic stress as metabolic triggers, researchers can redirect carbon flow to enhance pullulan output, viscosity, and purity for the production of structurally consistent, high-performance gels matrices.

### 2.3. Determinants of Molecular Weight and Material Properties

Elucidation of the genetic “blueprints” is fundamental, as the synchronized expression of specific genes and enzymes within *Aureobasidium* spp. directly dictates the fermentation efficiency, macromolecular weight, and chain architecture. While initiation of the pullulan chains requires specific α-1,4 primers as aforementioned the final molecular weigh is balanced between synthesis and enzymatic degradation [25]. Specifically, the molecular weight of pullulan is influenced by the secretion of pullulan-degrading enzymes, including α-amylase, glucoamylase, and pullulanase. The genetic inactivation of these hydrolases enables the production of pullulan with tailored molecular masses, allowing for the synthesis of ultra-high molecular weight variants. These are critical for manufacturing pharmaceutical drug capsules with superior stability [30] and sustainable food packaging films that can replace synthetic plastics like polyethylene due to their exceptional gas barrier properties and tensile resilience [31].

Beyond degradation, glycosyltransferases such as Gta1 modulate short α-1,4 glucan segments to influence the final chain length, while the presence of flexible α-1,6 linkages ensures an amorphous, highly soluble material [32]. These linkage patterns, in tandem with the molecular weight, determine the thermal decomposition range (250–300 °C) of the resulting polymer [10,33]. The final profile of pullulan-with respect of its mass, structural homogeneity, and degree-of branching-is a direct reflection of these underlying regulatory mechanisms. These biological controls govern functional behavior, including rheological viscosity, aqueous solubility, and tensile integrity. For instance, biosynthetic enhancement through the overexpression of *PUL1* can increase molecular weight of pullulan up to 4.4 × 10^5^ Da [15], whereas blocking degradation by knocking out the *amy1* gene can cause a 7.6-fold increase in mass by preventing chain cleavage. Furthermore, the secretion phase functions as a pivotal control point; the activity of the transmembrane transporter St1 has been shown to reduce molecular weight by 25%, potentially by altering cell membrane fluidity and secretion kinetics. Managing these physicochemical attributes allows the cellular machinery to produce a polysaccharide matrix suited for the structural requirements of high-performance functional gels [32].

Despite all the forementioned, pullulan biosynthesis can vary among *Aureobasidium* strains, resulting in substantial differences in pullulan yield, molecular weight distribution, melanin production, and responses to cultivation conditions. Consequently, genetic determinants identified in model strains may not always produce equivalent outcomes in industrial isolates. Furthermore, although key genes and regulatory mechanisms involved in pullulan biosynthesis have been elucidated, the transition into industrial-scale remains challenging due to strain-dependent physiological responses, the co-production of secondary metabolites, and the complex interplay between genetic regulation and fermentation parameters. These factors highlight the need for strain-specific optimization strategies to achieve consistent polymer quality and economically viable pullulan production [34].

## 3. Fermentation Environment: Tailoring Structural Quality and Yield

The industrial utility of pullulan is rooted in the unique physiology of its primary producer, *A. pullulans*. This resilient, polymorphous ascomycete is renowned for its ecological adaptability, thriving across environments ranging from plant surfaces and marine waters to nutrient-poor desert soils [33]. Due to its inherent metabolic flexibility, *A. pullulans* can produce various secondary metabolites, such as liamocins and enzymes [35]. Modern bioprocessing focuses on directing the organism toward its yeast-like morphology-the most productive state for pullulan synthesis, to minimize co-products and ensure the high purity required for GRAS compliance.

### 3.1. Nutritional Regulation of Pullulan Biosynthesis

The composition of the fermentation medium, specifically the selection and concentration of carbon and nitrogen sources, represents the most critical determinant of pullulan yield and quality. Production by *A. pullulans* and its related strains is highly sensitive to the nutritional environment, where the choice of substrates not only dictates the metabolic rate but also “programs” the final polymer’s molecular weight, purity, and cost-effectiveness. In the context of food-grade applications, these parameters are the primary drivers of gel strength and film integrity [13].

The type of sugar utilized acts as a metabolic blueprint for the resulting polysaccharide structure. While a wide array of carbon sources can be assimilated, sucrose and glucose remain the industrial benchmarks due to their high conversion efficiencies. Sucrose provides a robust supply of intracellular ATP and UDP-glucose precursors. However, its use can be complicated by the simultaneous formation of intermediate fructooligosaccharides, which may reduce polymer uniformity [36]. Glucose favors the assembly of polymers with higher molecular weight by suppressing the activity of hydrolytic enzymes that would otherwise degrade the nascent polysaccharide chains. While high glucose concentrations can inhibit production in wild-type strains, modern bioprocessing utilizing adaptive laboratory evolution has developed strains capable of reaching titers up to 162.3 g/L with high conversion efficiency (0.82 g/g) and productivity (1.13 g/(L∙h)) [37]. Substrates such as fructose, mannose, galactose, and xylose typically result in lower volumetric yields [19].

The concentration of the carbon source is a primary determinant of pullulan productivity, operating within a defined range of efficiency. For instance, increasing the glucose fraction from 2% to 4% has been shown to triple production, yet exceeding this 4% threshold typically induces a decline in titers. This is attributed to a combination of osmotic stress on the fungal cells, catabolite repression of biosynthetic genes, and potential product inhibition [8]. Similar inhibitory patterns are observed with sucrose and other complex sugars when concentrations exceed 5%, attributed to suppression of the enzymatic pathway responsible for pullulan assembly [38]. Furthermore, excessive carbon loading (>20%) can impair the physical environment of the fermenter by significantly increasing medium viscosity, which hinders agitation efficiency and restricts oxygen mass transfer [39]. However, certain agro-industrial substrates like corn bran hydrolysates have been successfully utilized at concentrations as high as 20% (*w*/*v*) before reaching inhibitory plateaus [40].

Nitrogen availability acts as the primary regulator of the transition between biomass accumulation and exopolysaccharide secretion. The onset of pullulan production is predominantly associated with the depletion or deliberate limitation of nitrogen in the culture medium. This nutritional stress serves as a critical physiological trigger, redirecting glucose metabolism away from cellular proliferation and toward the pullulan biosynthetic pathway. Nitrogen limitation enhances the activity of key intracellular enzymes, including α-phosphoglucomutase, glucosyltransferase, and UDPG-pyrophosphorylase, while simultaneously upregulating related biosynthetic genes (responsible for the polymerization and extrusion of the polysaccharide) such as *pgm1* and *fks* [41]. Excess of nitrogen, whether supplied through inorganic salts (ammonium sulfate, ammonium nitrate) or organic complexes (yeast extract and peptone), favors rapid biomass accumulation but significantly suppresses pullulan yield. Furthermore, elevated nitrogen levels in the late stages of fermentation can induce the activity of pullulan-degrading enzymes, leading to the undesirable hydrolysis of the polymer chains and a subsequent reduction in both molecular weight and total production [2]. While yeast extract is identified as the most effective organic source for high pullulan titers, its optimal thresholds should be determined to avoid repression of pullulan synthesis [42]. In industrial fermentations, ammonium sulfate and ammonium nitrate remain the most common inorganic nitrogen sources. However, the concentration threshold is critical since increasing the supply of ammonium sulfate to 7 g/L has been shown to shift the metabolic flux toward cell growth, thereby exhausting carbon resources that would otherwise be utilized for polysaccharide formation [43].

Ultimately, the successful production of high-purity pullulan for food-grade gels depends on maintaining a strategic carbon-to-nitrogen ratio. By ensuring that nitrogen becomes the limiting factor early in the production phase, the metabolic flux can be efficiently diverted to produce a consistent, high-molecular-weight polymer. This structural consistency is vital for the fabrication of active packaging and synergistic edible films that require high tensile strength and superior gas barrier properties.

### 3.2. Environmental Modulation

Maximum pullulan production is typically achieved at pH values between 6.5 and 7.5 and temperatures between 27 and 30 °C. Within these ranges, the organism maintains the productive yeast-like morphology necessary for growth and pullulan biosynthesis [44]. The mechanical environment, specifically oxygen availability, is equally vital. Agitation speeds (typically 200–700 rpm) and aeration rates (0.5–1.5 vvm) are essential to maintain appropriate dissolved oxygen levels (30–40%), which directly influence pullulan polymerization [45]. However, shear-induced mechanical degradation of the lengthening glucan chains significantly reduces the macromolecular entanglement density. This reduction in chain length and network complexity is detrimental to the structural integrity of the final material, leading to biomatrices with impaired tensile properties and diminished gas barrier efficiency. Specifically, a lower MW distribution limits the formation of a cohesive matrix, thereby increasing the free volume within the biomatric and compromising its capacity to exclude oxygen [46].

### 3.3. Supplementation Strategies

Beyond standard nutrients, the strategic addition of regulatory supplements can significantly enhance productivity and functional performance of pullulan. Mineral salts such as Fe^3+^, Mn^2+^, Zn^2+^, and Cu^2+^ modulate both the vegetative growth of *A. pullulans* and the kinetics of exopolysaccharide secretion. The effects of these ions are highly strain-specific; for instance, the addition of Cu^2+^ has been shown to enhance pullulan titers by 42.3%, likely through the stabilization of key biosynthetic enzymes. Conversely, Fe^3+^ and Zn^2+^ may exhibit inhibitory effects in certain cellular environments [47]. Also, adding 5 mM uracil post-48 h has been shown to increase yield by 30% by supporting the nucleotide sugar pool. Similarly, surfactants like Tween-80 improve secretion without proportionally increasing biomass [48]. All the aforementioned highlight the necessity of customized mineral profiles to maintain the productive yeast-like morphology and ensure enhanced fermentation efficiencies.

It should be noted that the effects of pH, temperature, oxygen availability, and nutrient composition are not independent. Variations in one parameter may influence the response to another through their combined effects on cellular metabolism, enzyme activity, morphology, and oxygen transfer. Consequently, optimal cultivation conditions are often strain- and process-specific and are commonly determined through multifactorial optimization approaches, such as response surface methodology, rather than by varying individual factors independently. This highlights the importance of considering process parameters as part of an integrated bioprocess system when interpreting pullulan production performance [49].

### 3.4. Renewable Feedstock Within the Circular Bioeconomy for Pullulan Production

**Figure 1:**
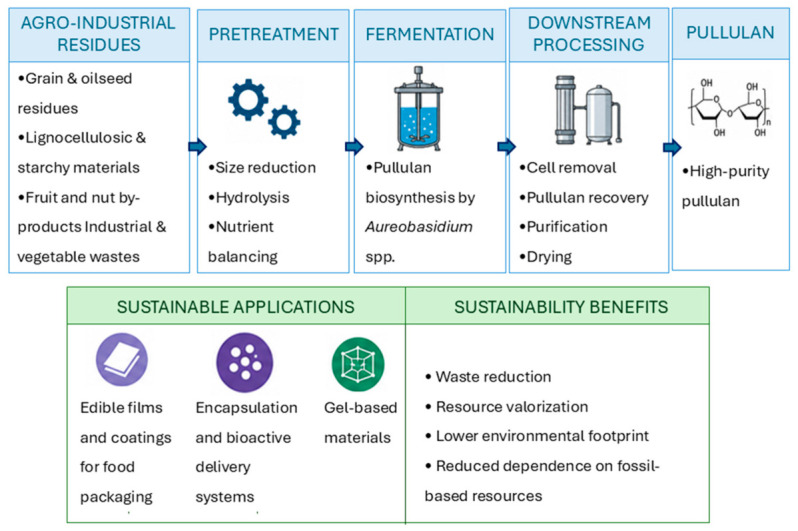
Circular bioeconomy approach for pullulan production and utilization.

The evolution of pullulan research has increasingly aligned with the principles of the circular bioeconomy, by replacing high-cost refined sugars with agro-industrial by-products [48]. This shift is driven by the fact that raw material costs can account for up to 50% of total production cost [48]. Consequently, low-cost residues derived from agricultural and food-processing industries have attracted considerable attention as alternative substrates capable of improving the economic feasibility and environmental sustainability of the bioprocess (Figure 1). Such feedstocks often contain micronutrients, minerals, and growth-promoting compounds that may stimulate the production of pullulan with distinct rheological profiles and molecular characteristics [37]. Moreover, their heterogeneous sugar composition and naturally occurring nutrients can support pullulan production with limited supplementation requirements [50].

*A. pullulans* exhibits remarkable metabolic versatility, utilizing diverse carbon sources and mixed sugar streams, e.g., glucose–xylose combinations commonly found in lignocellulosic hydrolysates [37]. The utilization of agro-industrial residues contributes to waste valorization, reduces disposal burdens, and promotes resource circularity. While they offer economic and environmental advantages over refined sugars, their greater compositional variability may affect process reproducibility and increase the need for pretreatment and process optimization. In contrast, commercial sugars provide a consistent composition and generally support higher pullulan concentrations, typically ranging from 101.4 to 125.7 g/L when combined with genetically optimized strains [51,52,53]. However, the reliance on food-grade substrates increases production costs and may limit the overall sustainability of the process.

A diverse array of biowastes has been successfully repurposed for pullulan biosynthesis. These feedstocks range from grain and oilseed residues (de-oiled rice bran), lignocellulosic and starchy materials (sugarcane bagasse, corn straw, corn cobs, and potato hydrolysates), fruit and nut by-products (apple pomace, almond hulls, citrus peels, grape pomace, hazelnut and chestnut hydrolysates, and banana peel extracts) to industrial and vegetable wastes (cheese whey, molasses, sugar beet pulp, pumpkin peel, melon and watermelon rinds, carrot peels, sweet potato, and onion waste) (Table 1).

A comparison of the studies presented in Table 1 reveals substantial variability in pullulan production depending on the feedstock, the producing strain, and the fermentation conditions employed. Among the investigated substrates, de-oiled rice bran supported the highest pullulan concentration (54.8 g/L), followed by apple pomace (38.4 g/L), almond hulls (34.3 g/L), and banana peel extract (33.7 g/L) [54,56,58,59]. Sugarcane bagasse-derived substrates led to pullulan concentrations ranging from 18.6 to 28.6 g/L, while corn-based feedstocks, including corn starch, corn cob, corn straw, and whole crop biomass, supported concentrations between 13.9 and 22.6 g/L [7,57,60]. In contrast, dairy by-products and mixed agro-industrial and vegetable residues, such as cheese whey, sugar beet pulp, melon rind, watermelon rind, onion waste, and carrot peels, generally resulted in lower pullulan production (5.0–14.5 g/L) [61]. The chemical composition of these substrates is an important factor influencing production outcomes, although the observed performance remains highly strain-dependent. A possible explanation for the aforementioned differences is the variation in nutrient availability among feedstocks. For example, de-oiled rice bran and apple pomace contain fermentable carbohydrates and naturally occurring nitrogen sources, minerals, and micronutrients that may contribute to enhanced pullulan production. In contrast, substrates such as cheese whey, melon rind, watermelon rind, and other vegetable-processing residues often possess lower carbon densities and higher moisture contents, which may partially explain their lower pullulan titers. These observations suggest that while such residues are attractive targets for waste valorization, their composition may limit pullulan accumulation unless appropriate supplementation or process optimization strategies are implemented.

The configuration of the fermentation system is another important determinant of process performance, as the cultivation environment influences the physiological state and metabolic activity of *Aureobasidium* strains. While shake-flask experiments remain indispensable for the preliminary screening of renewable feedstocks, they are limited by inefficient oxygen transfer, particularly as broth viscosity increases during pullulan accumulation. Transitioning to controlled cultivation systems, such as bubble column reactors or stirred-tank fermenters, can alleviate these limitations through the precise regulation of aeration and agitation rates [57,60]. However, scale-up does not inherently guarantee higher pullulan production. A notable example is *A. pullulans* LB83, which achieved a pullulan concentration of 25.2 g/L in shake flasks but only 18.6 g/L in a bubble column reactor [57]. Such findings indicate that reactor design and operating conditions must be carefully tailored to each strain–substrate combination. Therefore, the principal advantage of advanced bioprocessing systems lies not necessarily in achieving higher concentrations, but in maintaining stable and homogeneous cultivation conditions that promote process reproducibility and reliability, essential for industrial-scale implementation.

The integration of pullulan production within biorefinery frameworks represents a promising strategy for the valorization of agro-industrial residues and the transition toward a circular bioeconomy. By coupling microbial polysaccharide synthesis with regional waste management systems, agricultural by-products can be transformed into high-value biopolymers rather than being discarded, thereby improving resource efficiency and aligning production with global sustainability objectives [62]. The metabolic plasticity of *Aureobasidium* strains further enhances the feasibility of this approach. Consequently, the incorporation of pullulan biosynthesis into integrated biorefinery systems could reduce the environmental footprint of biopolymer production while supporting sustainable and decentralized bio-based manufacturing [60].

## 4. Downstream Processing: Purification and Recovery Strategies

The transition from a raw fermentation broth to a high-purity biopolymer requires a sophisticated sequence of separation and refinement. A significant challenge in pullulan production is the co-secretion of melanin, a dark, insoluble pigment that can compromise the transparency and esthetic appeal of food-grade films. The management of this issue begins at the biological level through the selection of non-pigmented strains, which inherently reduces the metabolic load and complexity of downstream purification [44]. When pigmented strains are utilized, the removal of melanin is a mechanical and chemical necessity. The process initiates with the removal of microbial biomass via centrifugation or microfiltration. The resulting cell-free supernatant is then treated with activated charcoal, the industrial standard for adsorbing residual melanin and low-molecular-weight impurities [38]. This step is vital for ensuring the transparent and colorless appearance demanded by the edible packaging industry or biomedical applications.

Once clarified, pullulan is recovered from the aqueous through fractional precipitation using short-chain alcohols such as ethanol, methanol, or isopropanol [63]. This technique exploits the differential solubility of the polysaccharide [38]. The efficiency of the recovery is highly dependent on the solvent-to-broth ratio and the temperature, which collectively influence the final molecular weight distribution of the polymer. Proper control during this stage is essential to ensure that the recovered pullulan retains the long-chain structure required for high tensile strength in functional food gels [63].

For applications requiring ultra-high purity, secondary refinement steps are implemented to reach food-grade or pharmaceutical-grade standards. Ion-exchange chromatography is utilized to eliminate residual proteinaceous matter and trace mineral salts that may interfere with the polymer’s cross-linking behavior or thermal stability. Furthermore, membrane-based techniques such as ultrafiltration and dialysis facilitate the removal of residual solvents and the concentration of the polymer while effectively narrowing its polydispersity index [38]. By managing these downstream strategies, manufacturers can “program” the purity and structural consistency of pullulan, ensuring it meets the performance benchmarks for targeted applications.

## 5. Physicochemical Resilience and of Pullulan

**Figure 2:**
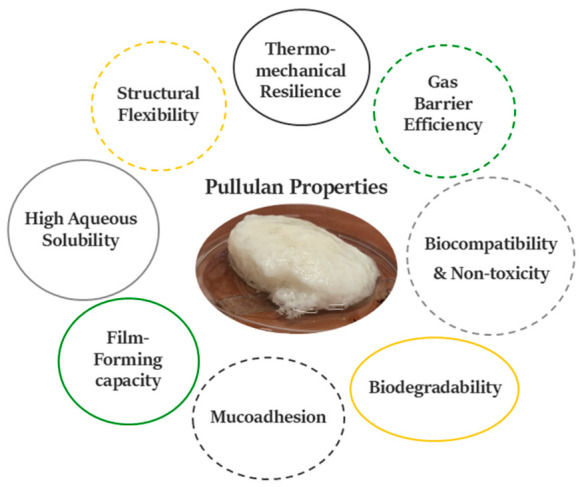
Key properties of microbial pullulan.

Pullulan is a linear, non-ionic, and hydrophilic exopolysaccharide that has emerged as a cornerstone biomolecule in food science and nutraceuticals. As a non-toxic, non-immunogenic, and non-mutagenic biopolymer, pullulan is recognized for its biocompatibility and biodegradability (Figure 2). These attributes have earned it the Generally Recognized as Safe (GRAS) designation for use across the food, pharmaceutical, and cosmetic sectors [64]. Pullulan is resistant to mammalian α-amylases, meaning it escapes standard enzymatic hydrolysis in the upper gastrointestinal tract. Consequently, it functions as a premium dietary fiber and prebiotic, selectively promoting the growth of beneficial *Bifidobacterium* species within the intestinal microbiota and contributing to overall digestive health [65].

**Figure 3:**
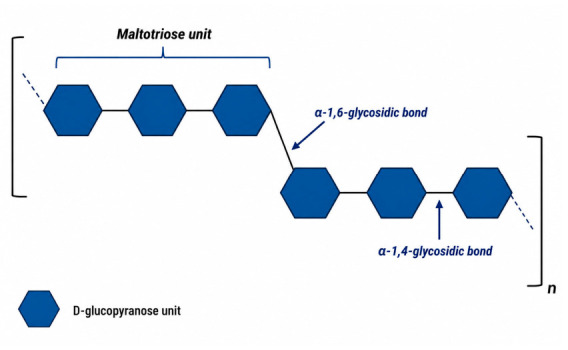
Chemical structure of pullulan.

The functionality of pullulan in food gel systems is attributed to its unique molecular architecture. The primary structure consists of linear α-D-glucan chains composed of repeating maltotriose units, where glucose residues are joined by α-1,4 glycosidic bonds. Successive maltotriose assemblies are interconnected via α-1,6 glycosidic bonds (Figure 3). This arrangement creates a ladder-like morphology that imparts significant segmental flexibility to the polymer backbone [66]. Such flexibility ensures the high aqueous solubility and its ability to undergo rapid hydration, which are essential for uniform hydrogel matrices. Unlike more rigid polysaccharides, the alternating bond pattern allows pullulan to maintain a random coil conformation in solution, providing the rheological versatility to stabilize complex food gels [9].

Compared with other polysaccharides commonly employed in food systems, pullulan exhibits a distinct balance of functional properties. Native cellulose generally provides superior mechanical strength and gas-barrier performance but requires chemical modification due to its limited solubility. Starch is abundant and inexpensive; however, its sensitivity to moisture and retrogradation can compromise stability during storage. Chitosan offers intrinsic antimicrobial activity but often requires acidic processing conditions and may produce brittle films. Similarly, alginate and pectin readily form gel networks through ionic interactions, although additional formulation strategies are frequently required to improve mechanical stability and water resistance. In contrast, pullulan is characterized by high aqueous solubility, excellent film-forming ability, and broad compatibility with other biopolymers. These characteristics make it particularly suitable for food systems, where complementary interactions with other polysaccharides can be exploited to achieve tailored material properties [1].

Building on the biological “blueprints” detailed in Section 2.3, the final molecular weight of pullulan, typically ranging from 40 kDa to 600 kDa, serves as the primary predictor of its film-forming capacity and gel strength [67]. This polydispersity is critical in food science; while low molecular weight fractions are ideal for rapid-dissolving oral films, the higher molecular weight polymer is essential for the robust, entangled networks required for cohesive biofilms and stable hydrogels [65]. As established previously, this macromolecular weight is not a fixed trait but a direct consequence of the fermentation environment, the specific microbial strain employed, and the complexity of the substrate. For instance, pullulan derived from sweet potato hydrolysate reaches a higher molecular weight compared to refined glucose or sucrose [68]. These nutrient-rich renewable hydrolysates may sustain the polymer chain elongation while preventing the rapid pH drops associated with simple sugar metabolism which can otherwise trigger enzymatic degradation by pullulanases [69].

From a thermal perspective, pullulan demonstrates remarkable stability, with decomposition typically initiating between 250 °C and 280 °C. This high threshold is a critical advantage for food-grade gels and biofilms that must undergo rigorous processing steps such as pasteurization or extrusion [49]. Its exceptional aqueous solubility across a wide temperature gradient facilitates the incorporation of bioactive compounds. To add, pullulan insolubility in most organic solvents ensures the structural integrity of pullulan-based matrices in lipid-rich food environments [70]. The rheological profile of pullulan is characterized by a stable viscosity that remains unaffected by pH fluctuations (2–10), temperature, or the presence of various metal ions [46]. However, pure pullulan lacks intrinsic, self-directed gelation due to its linear, non-ionic structure. The absence of charged functional groups along the glucan chain prevents the formation of strong electrostatic interactions or ionic cross-links. Consequently, neat pullulan tends to form rather weak, concentration-dependent entanglements [71].

## 6. Synergistic Gelation and Macromolecular Assemblies of Pullulan

The transition of pullulan from a versatile polysaccharide to a high-performance structural matrix is best realized through the mechanism of synergistic gelation, where its integration with various proteins and polysaccharides creates cohesive 3D networks with mechanical and rheological properties exceeding those of individual components. Pullulan acts as a high-density hydroxyl-functionalized scaffold that facilitates hybridized assemblies through localized hydrogen bonding and non-specific intermolecular interactions modulated by pH and ionic strength [65]. A defining feature of these assemblies is their structural continuity across hydration states; during dehydration, polymer chains align to form cohesive solid biofilms with favorable oxygen barrier properties, yet upon rehydration, the reactivation of hydrogen bonds allows the matrix to absorb up to 500% of its dry weight while reverting to a viscoelastic soft gel [72]. These systems exhibit controlled elasticity and pronounced thixotropy, a shear-thinning behavior where viscosity drops under mechanical stress and recovers upon resting, ensuring structural stability during storage while providing predictable flow dynamics during industrial processing [46]. This molecular flexibility allows pullulan/protein and pullulan/polysaccharides complexes to serve as a versatile backbone for chemical cross-linking or physical entanglement. To produce more stable gels, strategies like chemical cross-linking via dialdehyde-modified pullulan create stable covalent bonds between aldehyde and amino groups, while ionotropic gelation utilizes divalent metal ions like calcium or zinc to coordinate with hydroxyl groups and stabilize the structure [71]. These modifications significantly enhance the elastic modulus and water retention bridging the gap between simple film-forming solutions and complex, responsive food-grade hydrogels. Such versatility enables the engineering of fast-eroding gels and oral dissolving films that safeguard lipid-sensitive compounds or sensitive bioactives, providing up to 70% survival for encapsulated probiotics while ensuring rapid bioactive release and high bioaccessibility upon consumption [73].

### 6.1. Polysaccharide-Based Hybridized Networks

When integrated in matrices of guar gum or methylcellulose, pullulan drives the formation of cooperative assemblies that dictate aggregation temperature and overall gel strength. In methylcellulose-based systems it creates a loose fibrillar architecture that modulates thermal transition, allowing for finer control over the sol-to-gel transition [74]. In starch-dominated dough matrices, pullulan occupies the spaces between granules, enhancing water retention and ensuring textural stability throughout extended storage periods [75].

The inherent compatibility of pullulan with hydrophilic macromolecules, such as alginate and chitosan, enables the fabrication of high-performance biodegradable films through molecular-level integration. These interactions are rooted in interfacial compatibility; the abundant hydroxyl groups on the pullulan backbone engage with the amide groups of chitosan or the carboxyl groups of alginate, resulting in a homogenous polymer matrix that resists phase separation. This structural integrity is further enhanced through selective chemical modifications, such as oxidation. Oxidized pullulan serves as a potent crosslinking stabilizer for amino-rich polymers, utilizing Schiff base reactions between the aldehyde groups of the modified pullulan and the primary amines of the partner polymer. These hybridized blends produce hydrogels with tunable porosity, allowing for the precise engineering of pore dimensions to modulate diffusion rates in pharmaceutical delivery systems [76]. Beyond these porous networks, the synergistic assemblies exhibit enhanced mechanical and barrier properties critical for industrial applications. Alginate-pullulan blends, for instance, demonstrate a significant reduction in gas permeation compared to single-polymer films which is vital for active food packaging, as it extends the shelf life of sensitive lipid-based products [77]. In specialized pharmaceutical contexts, the synergy between pullulan and guar gum imparts unique mucoadhesive functionality. This interaction produces printable sublingual films characterized by high swelling indices and superior adhesion to mucosal tissues, facilitating efficient transmucosal delivery [78].

In complex multi-polymer hydrogel environments containing Poly(vinyl-alcohol), collagen, and chitosan, the integration of pullulan derivatives reinforces the mechanical topography of the 3D scaffold. This reinforcement increases the load-bearing capacity and structural integrity of the matrix, rendering it suitable for tissue engineering applications [79]. Furthermore, pullulan promotes nanofibrillar uniformity when incorporated into systems containing bacterial cellulose or cellulose nanofibers. In these composites, pullulan effectively coats the cellulosic filaments, promoting a more uniform distribution of hydrogen bonds and preventing nanofiber aggregation [80].

The role of pullulan in advanced nanotechnology highlights its versatility as both a structural matrix and a stabilizing carrier for bioactive encapsulation. In electrospinning processes, pullulan is blended with polysaccharides like starch and cellulose, or proteins such as zein, to stabilize the spinning jet and ensure the production of uniform, bead-free nanofibers. These hybridized nanofibers demonstrate enhanced thermal stability and can encapsulate hydrophobic bioactives-such as thymol-shielding them from environmental degradation while enabling a triggered release upon consumption [75].

A primary focus in recent research is the transition toward “green” synthesis, minimizing the use of organic solvents like DMF or DMSO in favor of purely aqueous systems for the fabrication of pullulan-based nanofibers. When reinforced with cellulose nanofibrils (CNF), the rheological behavior of these aqueous solutions is modulated by both the pullulan concentration and the solvent composition. Notably, increasing the CNF content from 1% to 5% has been shown to reduce fiber diameter from 284 nm to 90 nm, due to enhanced conductivity and increased surface charge density during the spinning process. These nanostructures, particularly when loaded with compounds like salicylic acid, exhibit zero-order release kinetics and exhibit potent antibacterial activity against both Gram-positive and Gram-negative bacteria, making them ideal candidates for high-performance drug delivery systems and functional food packaging [80].

### 6.2. Protein-Pullulan Synergies

Pullulan synergizes with a diverse array of proteins-including bovine serum albumin (BSA), whey protein isolate (WPI), ovalbumin, and plant-based proteins-to refine nanogel architecture and film mechanics. In ovalbumin-based systems, pullulan facilitates high-yield conjugation, often exceeding 80% efficiency via heat-induced rearrangements or Maillard reactions. These resulting nanogels provide superior encapsulation for hydrophobic compounds like curcumin, offering both steric stabilization and controlled release [38].

Blends of pullulan/proteins are primarily driven by pH-dependent electrostatic forces. For instance, at the isoelectric point of WPI (pH ≈ 5.2–5.5), the interaction between carboxyl and amino groups generates semi-crystalline networks with high elasticity storage modulus, and thermal stability, making these hybrids ideal for high-heat food processing [72]. In encapsulation applications, the pullulan/protein matrix functions as a dual-layered shield. The protein core provides hydrophobic pockets for sensitive bioactives like astaxanthin, curcumin or β-carotene, while the hydrophilic pullulan shell ensures environmental protection. This collaboration has been shown to triple the photostability of β-carotene and ensure up to 70% survival of encapsulated probiotics during simulated gastrointestinal transit [81].

Furthermore, pullulan functions as a steric barrier that limits protein aggregation during film casting, resulting in a homogenous microstructure essential for the predictable release of antimicrobial agents or bacteriophages [82]. This synergy is vital in nanotechnology; for proteins that typically lack the chain entanglement necessary for electrospinning-such as pea protein-the addition of pullulan provides the required viscosity and hydrogen-bonded network. While pullulan maintains the structural integrity of the spinning jet, the protein component provides functional value through improved bioaccessibility and nutrient-binding sites [83].

## 7. Pullulan Applications in the Food Industry

**Figure 4:**
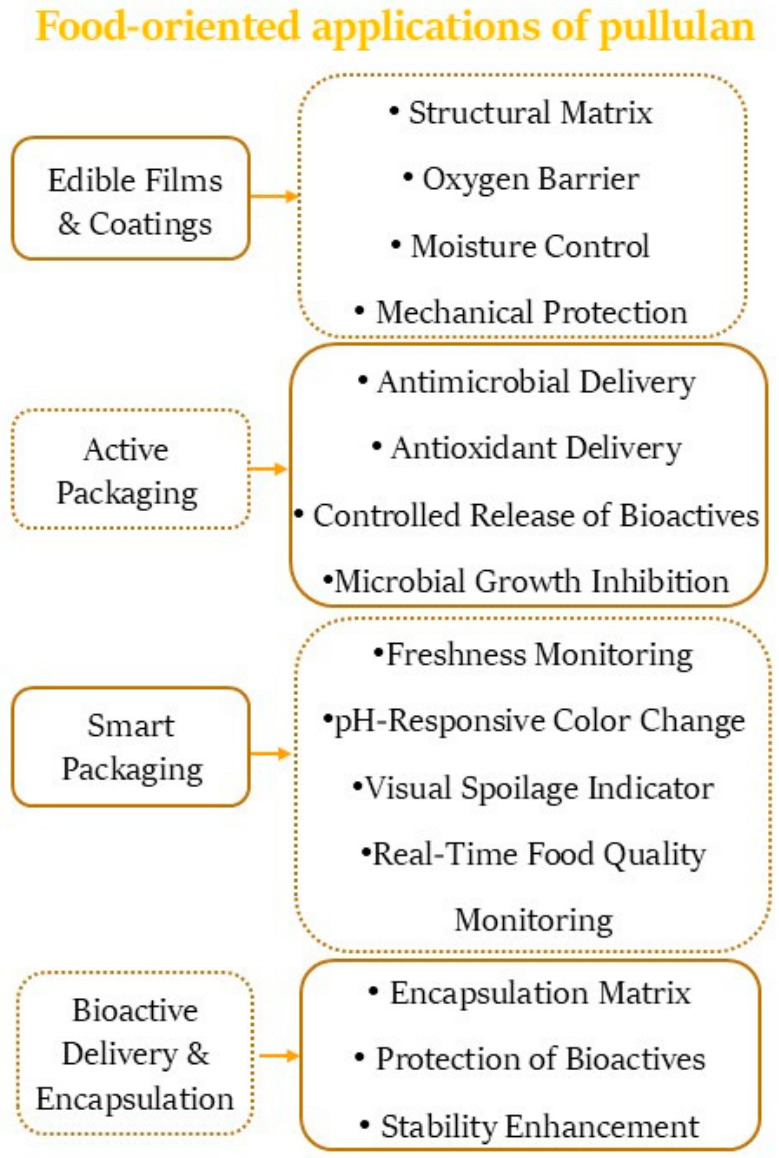
Multi-functional roles of pullulan in food-oriented applications.

The transition of pullulan from a laboratory-scale biopolymer to a versatile industrial material is driven by its GRAS status, biodegradability, excellent film-forming ability, and compatibility with a wide range of food-grade additives. Owing to its capacity to form cohesive, transparent, and mechanically stable matrices, pullulan has emerged as a promising platform for sustainable food systems (Figure 4). While production costs remain a limiting factor for widespread commercialization, ongoing developments continue to expand its functionality and application scope [65].

Among the available applications, edible coatings and films represent the most commercially feasible use of pullulan. Its linear molecular structure promotes dense molecular packing and extensive intermolecular hydrogen bonding, resulting in films with favorable flexibility, mechanical integrity, low oxygen permeability, and relatively low hygroscopicity [65]. Furthermore, pullulan forms compatible blends with biopolymers such as chitosan, pectin, and levan, allowing the development of composite structures without synthetic cross-linkers [73,84]. These properties have been successfully exploited for fresh produce preservation. Application of pullulan coatings to bananas, grapes, and strawberries has been reported to reduce moisture loss, delays ripening, and to limit microbial deterioration. For example, a formulation containing 10% (*w*/*v*) pullulan supplemented with calcium chloride and lemon juice increased banana firmness by ~55% while limiting weight loss to 5% over 20 days at 25 °C. The coating also reduced vitamin C degradation and maintained sugar content, highlighting its capacity to preserve both physical and nutritional quality [85]. Enhanced performance has also been achieved through chemical modification. Pullulan ester films exhibited remarkably low oxygen and water vapor permeability, enabling protection against oxidation and moisture migration. When applied to strawberries, these films effectively maintained firmness, titratable acidity, and soluble solids while extending shelf life compared with uncoated controls [86]. Due to their simplicity, scalability, and proven effectiveness, edible coatings constitute the most promising near-term application of pullulan.

Beyond its role as a passive barrier material, pullulan serves a carrier matrix for antimicrobial and antioxidant compounds, enabling the development of active packaging systems. In these formulations, pullulan combines its intrinsic barrier properties with the controlled release of bioactive substances that inhibit microbial growth and delay food spoilage. Pullulan matrices have been successfully used to encapsulate antimicrobial agents such as ε-polylysine, preserving their activity against foodborne pathogens. When applied to fresh fruits, these coatings function as semipermeable barriers that reduce respiration rates, moisture loss, and microbial contamination, thereby maintaining product firmness and overall quality [84]. The incorporation of inorganic antimicrobial nanoparticles further expands the functionality of pullulan films. Composite pullulan films containing silver and zinc oxide nanoparticles demonstrated strong inhibitory activity against *Listeria monocytogenes* and *Staphylococcus aureus* for up to 49 days. Similar systems effectively suppressed microbial proliferation in turkey deli meat during refrigerated storage, demonstrating their applicability to protein-rich foods with high spoilage susceptibility [87]. Additional functionality can be achieved through multilayer architectures, combining pullulan with complementary biopolymers such as chitosan, resulting in improved control of gas exchange and moisture transfer during storage. Such systems have successfully preserved vitamin C content, titratable acidity, and sensory characteristics in tropical fruits during post-harvest storage [88]. Although active packaging offers greater functionality than conventional coatings, large-scale implementation may be influenced by the cost, regulatory approval, and long-term stability of the incorporated bioactive compounds.

The most advanced application of pullulan lies in smart packaging technologies designed to monitor food quality in real time. The polymer’s compatibility with functional additives enables the incorporation of responsive compounds that generate detectable changes associated with food freshness. Recent studies have focused on pH-sensitive pullulan films containing natural indicators and bioactive ingredients such as cinnamon essential oil. These systems combine sensing capability with antimicrobial and antioxidant activity, creating multifunctional materials capable of both monitoring and protecting food products. Surfactants such as Tween 80 improve emulsion stability and prevent premature release of active compounds [89]. Other studies have expanded the functionality of pullulan-based intelligent packaging through the incorporation of natural colorimetric indicators. For example, pectin–pullulan composite films containing anthocyanin-rich hibiscus extract and tea tree oil-loaded Pickering emulsions demonstrated improved mechanical and barrier properties, enhanced antioxidant activity, and remarkable sensitivity to ammonia vapors. The films exhibited pronounced color changes in response to volatile spoilage compounds and successfully monitored the freshness of chicken meat in real time [90]. Similarly, smart films based on pullulan, xanthan gum, and κ-carrageenan enriched with cranberry anthocyanins showed excellent color responsiveness during fish storage. The indicator film changed from pink to yellowish-brown as fish spoilage progressed, correlating with increases in total volatile basic nitrogen and microbial counts [91]. These findings demonstrate the ability of pullulan-based intelligent packaging systems to provide visual and non-destructive assessment of food freshness while maintaining package integrity. Despite their significant added value, smart packaging systems remain at relatively low technology readiness levels. Challenges associated with sensor durability, the long-term stability of active agents and colorimetric indicators during storage, manufacturing complexity, and integration into existing packaging processes may influence the practical shelf life, reliability, and overall performance of pullulan-based packaging systems under real-world conditions [14].

Overall, the various applications of pullulan differ substantially in terms of technological readiness and commercial potential. Conventional edible coatings currently represent the most mature and economically viable option owing to their straightforward formulation and demonstrated effectiveness. They are particularly suitable for fresh fruits and vegetables where low-cost extension of shelf life is the primary objective. Active packaging systems provide greater functionality and are especially attractive for highly perishable foods requiring antimicrobial protection and prolonged storage stability. In contrast, smart packaging technologies offer the greatest innovation potential through real-time quality monitoring and are most relevant for high-value food products where freshness tracking and consumer information are critical. Consequently, while edible coatings are expected to dominate short-term commercialization efforts, active and smart packaging systems are likely to drive the next generation of high-value pullulan-based food packaging solutions [92].

## 8. Conclusions and Future Perspectives

Pullulan has emerged as a versatile microbial polysaccharide with significant potential for food-related applications due to its biodegradability, biocompatibility, film-forming ability, and compatibility with a wide range of proteins and polysaccharides. Recent advances in molecular biology have substantially improved our understanding of the genetic and regulatory mechanisms governing pullulan biosynthesis. The increasing utilization of agro-industrial residues has further strengthened the alignment of pullulan production with circular bioeconomy principles. Despite these advances, several challenges continue to limit large-scale implementation. The relationship between biosynthetic regulation, fermentation conditions, molecular architecture, and final material performance remains insufficiently understood. Although key genes and regulatory pathways have been identified, predictive patterns between microbial metabolism, polymer structure, gel-forming behavior, and application-specific functionality have yet to be established. Addressing this knowledge gap would facilitate the transition from empirical process optimization toward the rational design of pullulan-based materials with tailored physicochemical properties.

Future research should focus on the development of robust, high-yield, and low-pigment-producing strains through systems biotechnology, metabolic engineering, and multi-omics-guided approaches. In particular, strategies that redirect carbon flux toward pullulan biosynthesis while minimizing the formation of undesired by-products, may significantly improve process efficiency and reduce downstream purification requirements. At the same time, the utilization of region-specific agro-industrial residues should be further explored. Emphasis should be placed on managing feedstock variability and ensuring consistent polymer quality, molecular weight distribution, and rheological performance. From a materials perspective, future developments are expected to move beyond conventional pullulan systems toward multifunctional hybrid materials. The incorporation of nanostructured reinforcements and complementary biopolymers may enable the development of advanced hydrogels, active packaging, and intelligent packaging systems with improved mechanical performance, barrier properties, and controlled-release functionalities. Furthermore, techno-economic and life cycle assessment should be incorporated from the earliest stages of process development to evaluate industrial feasibility and environmental sustainability.

Ultimately, the successful industrial implementation of pullulan will depend on the integration of microbial biotechnology, bioprocess engineering, material science, techno-economic assessment, and sustainability evaluation. Beyond its direct industrial relevance, pullulan also represents an attractive model system for understanding how microbial biosynthesis influences polymer architecture and material functionality. Such interdisciplinary approaches may support the development of next-generation pullulan-based systems, advancing the broader field of sustainable bio-based materials. By synchronizing biosynthetic efficiency with material performance and sustainability considerations, pullulan has the potential to become an important component of future bio-based food, packaging, and functional material technologies.

## Figures and Tables

**Table 1:** Pullulan production by *Aureobasidium pullulan* strains using agro-wastes as nutrient sources.

Strain	Renewable Feedstock	Pullulan (g/L)	Fermentation Type/Conditions	Productivity g/(L∙d)	References
*A. pullulans* MTCC 1991	Apple pomace	38.4	Shake flasks	5.5	[54]
*A. pullulans* AZ-6	Citrus peels, grape pomace, hazelnut and chestnut hydrolysates, sugarcane molasses, pumpkin peel	7.4–33.6	Shake flasks	~0.9–4.2	[55]
*A. pullulans* MTCC 6994	de-oiled rice bran	54.8	Shake flasks	7.8	[56]
*A. pullulans* LB83	Sugarcane bagasse hydrolysate	25.2	Shake flasks	6.7	[57]
		18.6	Bubble column bioreactor	4.6	
*A. pullulans* KY767024	Almond hulls	34.3	Shake flasks	4.9	[58]
*A. pullulan* MTCC 2195	Banana peel extract	33.7	Shake flasks	-	[59]
*A. pullulans* CCTCC M 2012259	Corn starch/Corn cob/Corn straw	21.8/13.9/14.0	5 L fermenter	7.3/4.6/4.7	[60]
Evolved strain EV6	Corn starch/Corn cob/Corn straw/Whole crop	22.6/21.7/20.2/21.9		7.5/7.2/6.7/7.3	
*A. pullulans* ATCC 42023	Sugarcane bagasse hemicellulosic hydrolysate	28.6	Bubble column reactor	5.7	[7]
*A. pullulans* AZ-6	Cheese whey, molasses, grape pomace, sugar beet pulp, melon rind, watermelon rind, onion waste, carrot peels, sweet potato	5.0–14.5	Shake flasks	-	[61]

## Data Availability

No new data were created or analyzed in this study. Data sharing is not applicable to this article.
